# Biologically Relevant Micellar Nanocarrier Systems for Drug Encapsulation and Functionalization of Metallic Nanoparticles

**DOI:** 10.3390/nano12101753

**Published:** 2022-05-20

**Authors:** Victoria Valdivia, Raúl Gimeno-Ferrero, Manuel Pernia Leal, Chiara Paggiaro, Ana María Fernández-Romero, María Luisa González-Rodríguez, Inmaculada Fernández

**Affiliations:** 1 Departamento de Química Orgánica y Farmacéutica, Facultad de Farmacia, Universidad de Sevilla, 41012 Seville, Spain; rgimeno@us.es (R.G.-F.); chiara.paggiaro@studenti.units.it (C.P.); 2 Departamento de Farmacia y Tecnología Farmacéutica, Facultad de Farmacia, Universidad de Sevilla, 41012 Seville, Spain; anaferrom2@alum.us.es (A.M.F.-R.); malugoro@us.es (M.L.G.-R.)

**Keywords:** micelles, drug encapsulation, metallic nanoparticle functionalization

## Abstract

The preparation of new and functional nanostructures has received more attention in the scientific community in the past decade due to their wide application versatility. Among these nanostructures, micelles appear to be one of the most interesting supramolecular organizations for biomedical applications because of their ease of synthesis and reproducibility and their biocompatibility since they present an organization similar to the cell membrane. In this work, we developed micellar nanocarrier systems from surfactant molecules derived from oleic acid and tetraethylene glycol that were able to encapsulate and in vitro release the drug dexamethasone. In addition, the designed micelle precursors were able to functionalize metallic NPs, such as gold NPs and iron oxide NPs, resulting in monodispersed hybrid nanomaterials with high stability in aqueous media. Therefore, a new triazole-derived micelle precursor was developed as a versatile encapsulation system, opening the way for the preparation of new micellar nanocarrier platforms for drug delivery, magnetic resonance imaging, or computed tomography contrast agents for therapeutic and diagnostic applications.

## 1. Introduction

In recent decades, surfactants have been widely used in the development of new functional nanostructures with potential applications in different research areas, such as electronics, solar cells, batteries and biomedicine [1,2,3], among others. In fact, their applications in the field of biomedicine currently represent great promise for targeted delivery, improved bioavailability, and controlled drug release [4,5,6,7,8,9,10]. Surfactants are organic molecules that are capable of organizing themselves, in aqueous media, into different types of supramolecular structures such as micelles, monolayer vesicles (also known as liposomes), bars, sheets and tubes [11]. The most illustrative example of this kind of supramolecular organization is the cell membrane of living cells, a complex nanosystem formed by units of phospholipids that organize as a bilayer structure. Among the different types of supramolecular structures, micelles have attracted great attention as excellent nanosystems for biomedical applications [12]. Micelles are spheroidal structures with a hydrophilic shell and a hydrophobic core with sizes between 10 and 200 nm that provide them with high stability under physiological conditions and bioavailability. Thus, micelles could be less phagocytosed by the mononuclear phagocyte system compared to those structures with larger dimensions [13,14]. Moreover, micelles in this size range could be an interesting nanoplatform for in vivo testing without risk of blood vessel blockage. Furthermore, the presence of a hydrophobic inner part in their structure provides micelles with the ability to encapsulate non-water-soluble molecules, acting as excellent nanosystems to carry drug molecules or other payloads such as contrast agents, for instance.

Therefore, taking all of these features into account, micelles could be used as drug nanocarriers in passive targeting by the enhancement of permeability and retention effect [15], or even in active targeting in the case of functionalized micelles with appropriate vectors at their surface that direct drugs towards the specific cell, tissue or organ. For example, vectors such as carbohydrates [16], folic acid [17], antibodies [18], proteins [19], peptides [20] and aptamers [21] have been used for this purpose.

On the other hand, micelles could also be used as imaging agents by encapsulating the corresponding contrast agents in their inner hydrophobic cores. In this sense, various agents have been encapsulated for biomedical imaging techniques, such as metallic nanoparticles [22,23]. These metal NPs are receiving interest due to enhancements in the resolution and sensitivity of the most common techniques available in routine clinical practice, such as computed tomography (CT) and magnetic resonance imaging (MRI) [24,25].

**Figure 1:**
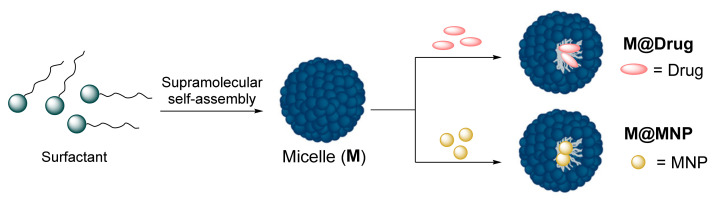
Formation of micellar nanocarrier systems by self-assembled supramolecular surfactants followed by encapsulation of drugs or metallic NPs (MNPs). (M@Drug = micelles containing the drug. M@MNP = micelles containing the MNPs).

In this work, we report the preparation of different micellar nanocarrier systems based on a surfactant molecule derived from oleic acid and tetraethylene glycol for drug delivery, using dexamethasone (Dexa) as a drug model, and for functionalization of metallic NPs such as gold NPs and iron oxide NPs. These nanosystems are expected not to show any important sign of cytotoxicity due to the structural similarity of our designed micelles to other reported nanocarriers that showed an absence of toxicity after their exposure to cultured cells and also mice, as previously published elsewhere [26,27]. Moreover, the presence of metallic NPs such as AuNPs and IONPs would not be a safety issue, as already reported in an exhaustive toxicity screening of water-soluble NPs on cell cultures, zebrafish embryos and mice [28]. Furthermore, these nanosystems could be used as potential therapeutic and/or diagnostic agents for different biomedical applications. (Figure 1).

## 2. Materials and Methods

All reagents and solvents were obtained from commercial suppliers (Merck (Darmstadt, Germany) and Fisher Scientific (Waltham, MA, USA), including iron chloride (III), sodium oleate, oleic acid 99%, 1-octadecene, tetraethylene glycol (TEG), triethylamine, 4-dimethylaminopyridine, diisopropyl carbodiimide (DIC), hydrochloric acid (HCl), and sodium sulfate (Na_2_SO_4_). Solvents such as toluene, ethanol, acetone, hexane, chloroform, dichloromethane and tetrahydrofuran were of anhydrous and high performance liquid chromatography (HPLC) grade. Organic synthesis monitoring was performed by thin layer chromatography (TLC) on a sheet of aluminum coated with silica gel 60 F254 purchased from Merck (Darmstadt, Germany). The TLC results were revealed with a solution of 5% phosphomolybdic acid in EtOH. Silica gel 60 (0.04–0.063 mm) from Merck (Darmstadt, Germany) was used for flash chromatography.

### 2.1. Characterization Methods

− Nuclear magnetic resonance (NMR) spectra were recorded on a BRUKER AMX-500 apparatus (Billerica, MA, USA). Deuterated chloroform, water, dimethylsulfoxide and methanol were used and indicated in parentheses for each compound. The chemical shift values (δ) were referred to tetramethylsilane used as an internal reference.
− High resolution mass spectrometry (HRMS) was recorded on a Q Exactive Hybrid Quadrupole-Orbitrap apparatus from ThermoFisher Scientific (Waltham, MA, USA).
− Transmission electron microscopy (TEM) images were obtained on an HR Fei Talos 200X microscope (Waltham, MA, USA) operated at an accelerating voltage of 100 kV. TEM samples were prepared by dropping a solution of the corresponding nanosystem onto a carbon-coated copper grid without staining. The mean sizes were calculated on an average of 100 nanoparticles measured.
− The intensity distribution of the nanosystems was measured on a Zetasizer Nano ZS90 from Malvern Panalytical (Malvern, UK). The dynamic light scattering (DLS) measurements were performed on a cell type: ZEN0040 disposable cuvette, setting a refractive index of 2.3 for iron oxide NPs, 0.2 for the AuNPs and 1.40 for the micelles without metallic NPs. The measurement duration was set as automatic and 3 as the number of measurements. As analysis model, the general purpose (normal resolution) was chosen. The nanosystems were suspended in the corresponding solvent at a concentration of 1 mg/L of micelles and/or metallic NP.
− HPLC was performed on a reversed phase column ZORBAX Eclipse XDB-C18, Narrow-Bore 2.1 × 150 mm, 3.5 Micron from Agilent (Santa Clara, CA, USA). The elution was carried out using water, 0.1% FA (A)-methanol, 0.1% FA (B) with the following elution gradient: 20% B (0.5 min), linear gradient from 20 to 100% of B for 11.5 min, isocratic gradient to 100% of B (1.5 min) and return to initial conditions (20% of B) for up to 15 min with constant flow of 0.3 mL/min and injection volume of 10 µL.

### 2.2. Encapsulation and Release of Dexamethasone

Dexa encapsulation was performed following this procedure: (i) Direct addition of the solid drug to the previously prepared micelle water solution and stirring for 72 h at 50 °C. During this time, the sample was covered with aluminum foil to prevent the degradation of the photosensitive drug Dexa. (ii) Centrifugation at 2000 rpm for 15 min, obtaining a precipitate, which represented the drug not included, and a solution containing the micelles with the internalized drug, and (iii) lyophilization of the aqueous solution.

Micelles loaded with Dexa (named as **M3@Dexa** and **M5@Dexa**) (2 mg) were transferred to a dialysis bag containing 4 mL of milliQ water at pH 7.4. This bag was then placed in a beaker with 300 mL of milliQ water at pH 7.4 and gently stirred for 52 h at room temperature. The bag was fully immersed under the surface of the liquid. Aliquots of about 2 mL were withdrawn from the 300 mL recipient at specified time periods. The withdrawn aliquots were replaced with equal volumes of milliQ water. The aliquots were then filtered using a 45 μm syringe filter and analyzed using HPLC.

### 2.3. Synthesis of Iron Oxide NPs

The synthesis of iron oxide NPs (IONPs) was prepared following a previously published procedure with slight modifications [29]. Briefly, the iron precursor (iron oleate) (3.6 mmol), oleic acid (1.8 mmol) and 20 g of 1-octadecene were heated to 320 °C for 1 h in a three-necked round-bottom flask under a flow of argon. The mixture was then cooled to room temperature. Finally, IONPs were washed with ethanol–acetone (1:1) to remove subproducts, then centrifuged. The IONPs were suspended in toluene.

### 2.4. Synthesis of Au NPs

For the preparation of gold NPs, the method published by Swihart and colleagues was used [30]. Briefly, a suspension of the precursor HAuCl_4_·3H_2_O in oleylamine (1 mL) was added to oleylamine (5 mL) at 150 °C in a three-necked round-bottom flask under argon atmosphere. The mixture was heated for 1.5 h. Next, the mixture was cooled down to room temperature, and the NPs were washed with ethanol as precipitation agent and centrifuged. Finally, the AuNPs were suspended in hexane.

### 2.5. Functionalization of Metallic NPs

Metallic NP functionalization with the micelles was performed in a glass vial by sonicating with a probe the ligand **3** (5 mg) and the corresponding solution of NP (250 μg of Au or Fe) in 5 mL of water. The resulting mixture was filtered through a 0.2 μm pore size syringe filter (membrane material: acrylic copolymer housing cellulose acetate/surfactant-free membrane), and the filtered solution was dialyzed against water for 2 days using a membrane (MWCO: 50 kDa).

## 3. Results and Discussion

**Figure 2:**
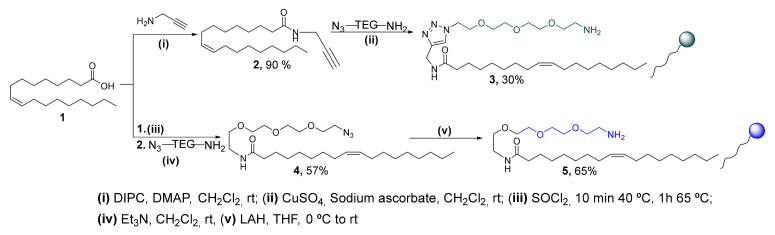
Synthesis of surfactants **3** and **5** by a two-step synthetic route.

The preparation of the micellar nanocarrier systems was carried out independently from two different surfactants derived from oleic acid as the lipophilic tail and an amino-ended tetraethylene glycol (TEG) spacer as the polar head [31]. Both surfactants were prepared in a two-step synthetic route as shown in Figure 2. On the one hand, the triazole derivative surfactant **3** was synthesized by amidation between oleic acid and propargylamine followed by a Cu (I) catalyzed Huisgen reaction with the N_3_-TEG-NH_2_ spacer [32]. On the other hand, the amide derivative surfactant **5** was prepared by linking oleic acid with the N_3_-TEG-NH_2_ spacer in a two-step reaction. First, the oleyl chloride, generated in situ by treating oleic acid with SOCl_2_, was added directly to the spacer, yielding the corresponding amide **4**. Then, the azido group present in **4** was reduced to amine with LAH, rendering the surfactant **5** in good yield.

Both surfactants present an amino group at the polar head that could play important roles. For instance, the micelles and also the micelle formations could be affected by the pH conditions due to the change in the hydrophilic/lipophilic balance of the surfactant. So, these amino groups could confer to the micelles advantages such as pH-responsive properties [33] and versatility by allowing the incorporation of active targeting ligands on their surfaces.

The only difference between surfactants **3** and **5** was the connector linking the hydrophobic tail and the polar head, a triazol and an amide group, respectively. However, these connectors did not show significant differences in the resulting micelles, as our group reported in a previous work [31]. Therefore, the surfactants **3** and **5** generated the micelles **M3** and **M5**, respectively, in water at a slightly acid pH (pH 6), at a concentration much higher than their corresponding critical micellar concentration (CMC), 0.05 mM and 0.07 mM for M3 and M5, respectively (see supporting information).

### 3.1. Loading and Drug Release

**Figure 3:**
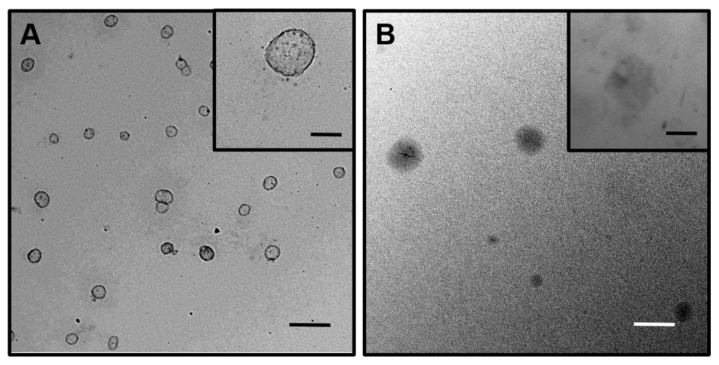
Representative TEM images of: (**A**) **M3@Dexa** and (**B**) **M5@Dexa**. Scale bars correspond to 200 nm for low magnification TEM images and 20 nm for the insets.

In order to determine the usefulness of these micelles as drug nanocarrier platforms, Dexa was chosen as a lipophilic model drug. Dexa is a well-known anti-inflammatory corticosteroid commonly used to treat different pathologies such as inflammation, arthritis and allergic reactions, among others [34]. Additionally, it has recently shown its effectiveness in the treatment against SARS-CoV-2 in adults and adolescents over 12 years of age with pneumonia that require oxygen [35]. Thus, we decided to carry out Dexa encapsulation in both synthesized micelles **M3** and **M5**. In order to confirm the inclusion of Dexa in the micelles, ^1^H NMR spectra were recorded in different deuterated solvents as D_2_O, methanol-d4 and dimethylsulfoxide-d6 (spectra shown in the supporting information), and the percentage of internalization was determined by gravimetry. In both micelles, Dexa was encapsulated with good percentages, 54.2% and 74.2% for **M3** and **M5,** respectively. The difference in cargo capacity among the micelles could be attributed to the different connectors included in their structures. In the case of **M3**, the triazol connector conferred on the surfactant an increase in hydrophobicity compared to the amide connector in **M5**. However, in **M5,** intermolecular hydrogen bonds were favored. This variation in the hydrophilic/hydrophobic balance led to remarkable differences in the mean size of both micelles, with values of 98.5 and 190 nm for the **M3** and **M5,** respectively, as determined by DLS, explaining their different capacities of internalization. Moreover, the characteristic size and morphology of the micellar nanocarrier systems containing Dexa were determined by TEM (Figure 3A,B).

**Figure 4:**
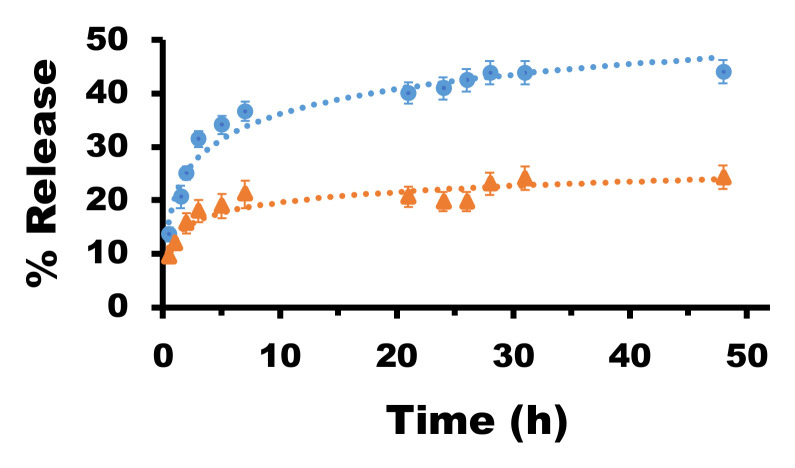
Drug release studies with **M3@Dexa** (blue circle) and **M5@Dexa** (orange triangle).

To investigate the drug release of the nanocarrier systems, the Dexa-loaded micelles **M3@Dexa** and **M5@Dexa** were incubated at 25 °C under physiological conditions in a dialysis membrane with a molecular weight cut-off of 10 kDa. After certain time lapses, a volume of the solution containing the released Dexa from the carriers was measured by HPLC. The data points were collected until the graph reached a plateau. These data from both nanocarriers showed a different behavior of the released Dexa. The release profile of the drug from the micelles in the dialysis membrane presented an initial burst release of 25% and 15% in the case of **M3@Dexa** and **M5@Dexa,** respectively. After 48 h, the **M3** micelles showed a higher release of Dexa than the **M5** micelles, of 45% and 25%, respectively. These results clearly indicated a stronger interaction of the nanocarrier **M5** with Dexa than the **M3**, limiting the leakage of the drug from the inner part (Figure 4). The presence of a triazol group in the **M3** likely reduced the interactions of the lipidic tail with the drug Dexa, thus generating a less stable system that facilitated the release of Dexa.

Additionally, since the micelles displayed amino groups to the medium, the pH effect on the hydrodynamic (HD) size was also studied by DLS. **M3@Dexa** was selected for these studies, due to its smaller size than that of **M5@Dexa,** which made it more interesting for future in vivo applications. At basic pH, we observed an increase in the HD size to 885 ± 182 nm with a high polydispersity index (0.6) that indicated the presence of polydisperse particles (Table 1). Interestingly, at acid pH, the micelles presented an HD size of 43.1 ± 0.8 nm with a quite low PDI value of 0.38. This behavior might be explained by the complete protonation of the amine functional groups at acid pH. The amino groups could be completely charged, inducing an increase in the hydrophilia of the micelles with the corresponding decrease in HD size. Contrarily, the amino groups at basic pH were completely uncharged, reducing the polarity of the system and thus rendering micellar aggregation.

### 3.2. Functionalization of Metallic NPs

To investigate the capacity of our micelles to encapsulate inorganic nanoparticles for potential biomedical applications, **M3** micelles with HD sizes smaller than 100 nm were chosen for this purpose. The functionalization of the NPs was performed in a one-step process, which meant that, as in the encapsulation of the Dexa drug, the formation of the micelles before the addition of the NPs was not required. Thus, a solution of surfactant **3** in water at a higher concentration than CMC was mixed with a suspension of metallic NPs in hexane for the AuNPs and toluene for the IONPs, respectively. The mixture was then sonicated with a sonication probe for 30 min, resulting in the transfer of the metallic NPs to the aqueous medium. The functionalized NPs were purified by following two different methods. First, the mixture was filtered through a 0.2 μm pore size syringe filter to remove the large aggregates formed during the functionalization. The NP suspension was then dialyzed in dialysis tubing against water to remove the free surfactants that could be in the solution. Therefore, the obtained **M3** micelles were able to functionalize two different metallic NPs as IONPs and AuNPs, resulting in the new nanomaterials **M3@IONPs** and **M3@AuNPs**, respectively.

### 3.3. Characterization of the Functionalized Metallic NPs

**Figure 5:**
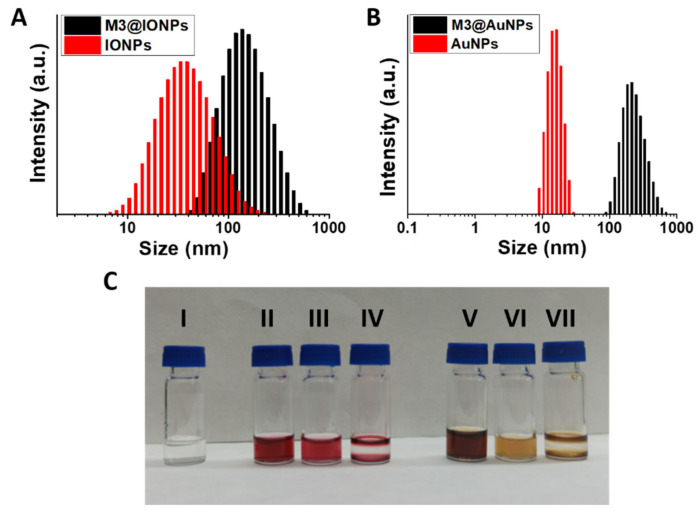
(**A**) Intensity size distributions of **M3@IONPs** in water (black) and IONPs in toluene (red). (**B**) Intensity size distributions of **M3@AuNPs** in water (black) and AuNPs in hexane (red). (**C**) Picture comparing the nanomaterials in suspension: (I) **M3** in water, (II) AuNPs in hexane, (III) **M3@AuNPs** in water, (IV) AuNPs in water, (V) IONPs in toluene, (VI) **M3@IONPs** in water and (VII) IONPs in water.

These nanomaterials were first characterized by DLS, measuring their HD sizes in water (Figure 5A,B, and Table 2). As shown in Figure 5A, the intensity size distribution of the IONPs showed a substantial increase from the oleic acid capped IONPs (insoluble in water) to the IONPs encapsulated micelles (water soluble). In the same way, AuNPs showed a remarkable difference in the intensity size distribution from the oleyl amine capped AuNPs to the AuNP encapsulated micelles (Figure 5B). An increase in the HD sizes was observed in both functionalizations, resulting diameters of 101.6 ± 1.4 nm and 240.7 ± 7.4 nm for the **M3@IONPs** and **M3@AuNPs,** respectively (Table 2). It is also important to note that the encapsulation of metallic NPs renders quite stable and monodisperse particles, as pictured in Figure 5C with polydispersity indexes of 0.18 and 0.25 for the magnetic and gold derived micelles, respectively (Table 2 and Supplementary Materials).

**Figure 6:**
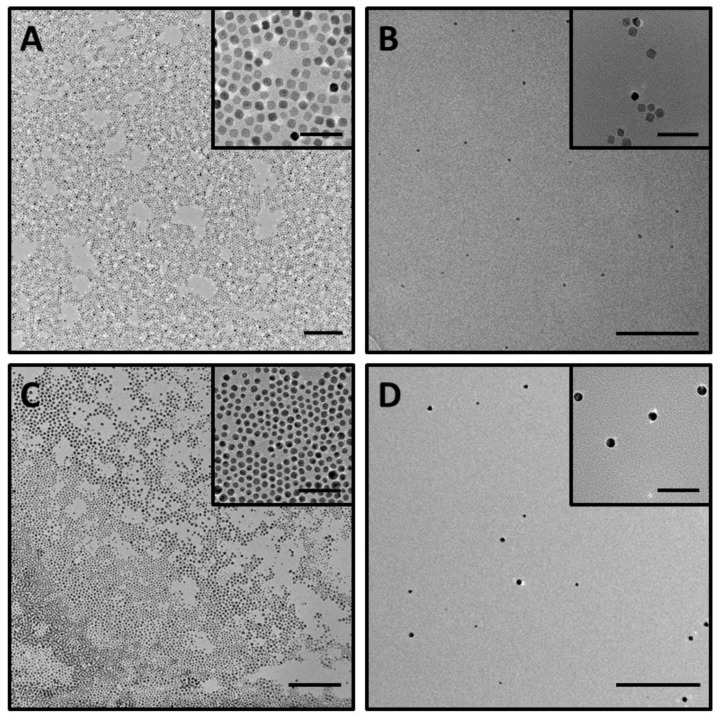
Representative TEM images of: (**A**) IONPs in toluene, (**B**) **M3@IONPs** in water, (**C**) AuNPs in hexane and (**D**) **M3@AuNPs** in water. Scale bars correspond to 200 nm for the low magnification TEM images and 50 nm for the insets.

Finally, to demonstrate the presence of metallic NPs in the solutions, TEM images of the corresponding micelles with encapsulated NPs were analyzed (Figure 6). In both nanomaterials, the functionalized IONPs and AuNPs were observed to be homogeneously distributed on the grid without the presence of any aggregates. However, the presence of the organic component of the micelles was not observed in these TEM images because of the high contrast from the inorganic NPs.

## 4. Conclusions

In this work, we developed a versatile micellar nanocarrier system able to encapsulate a hydrophobic drug such as Dexa and also metallic NPs such as IONPs and AuNPs. These micellar systems are based on surfactants derived from oleic acid as the hydrophobic tail and the amino-ended TEG spacer as the polar head. Moreover, the cargo encapsulation and release were shown to depend on the nature of the groups that connect the polar head and the hydrophobic tail, thus encapsulating 54% and 74% of the drug cargo for the triazol and amide connector derived micelles, respectively. On the other hand, the release profiles at physiological pH showed that the **M3** micelles with a triazol connector allowed a higher percentage of free Dexa than the **M5** micelles with an amide connector. Furthermore, the **M3** micelles were also capable of functionalizing metallic NPs as IONPs and AuNPs, thus resulting in hybrid nanomaterials with high stability and monodispersity in aqueous medium. To conclude, great versatility of encapsulations has been achieved by using the micellar precursor **3,** providing access to new micellar nanocarrier platforms for drug delivery, magnetic resonance imaging, or computed tomography contrast agents for therapeutic and diagnostic applications.

## Figures and Tables

**Table 1:** Hydrodynamic size of **M3@Dexa** at different pHs.

M3@Dexa	Size (nm)	Std. Dev.	PDI
pH 10	884	182.3	0.6
pH 7	98.5	1.6	0.37
pH 3	43.1	0.8	0.38

**Table 2:** HD sizes with the corresponding polydispersity indexes of the different nanomaterials.

	Size (nm)	PDI	Std. Dev.
**M3@AuNPs**	240.7	0.25	7.4
**M3@IONPs**	101.6	0.18	1.4
AuNPs	14.6	0.01	0.04
IONPs	29.9	0.22	0.8

## Data Availability

The data presented in this study are available on request from the corresponding author.

## Supplementary Materials

The following supporting information can be downloaded at: https://www.mdpi.com/article/10.3390/nano12101753/s1. Figure S1: ^1^H-NMR of 3 in D_2_O; Figure S2: ^13^C-NMR of 3 in MeOD; Figure S3: ^1^H-NMR of 5 in D_2_O; Figure S4: ^13^C-NMR of 5 in MeOD; Figure S5: HRMS Spectrum of 3; Figure S6: HRMS Spectrum of 5; Figure S7: Top graph corresponds to HPLC chromatogram of 3. Bottom graph corresponds to MS Spectrum of 3; Figure S8: Top graph corresponds to HPLC chromatogram of 5. Bottom graph corresponds to MS Spectrum of 5; Figure S9: ^1^H-NMR of dexamethasone in DMSO-d_6_; Figure S10: ^1^H-NMR of M3 in D_2_O; Figure S11: ^1^H-NMR of M5 in D_2_O; Figure S12: ^1^H-NMR of **[M3@Dexa]** in D_2_O (a) and in DMSO-d_6_ (b). ^1^H-NMR of **[M5@Dexa]** in D_2_O (c) and in DMSO-d6 (d). In pink are indicated the new signals that appear in DMSO-d6 ((b) and (d)) corresponding to dexamethasone; Figure S13: CMC curve of Compound **3**; Figure S14: CMC curve of Compound **5**; Figure S15: (A) HPLC chromatogram of Dexa, (B) UV absorption spectrum of Dexa, and (C) Calibration curve of Dexa; Figure S16: Hydrodynamic sizes of micelles **M3**, **M3@IONPs** and **M3@AuNPs** in water vs. time.
