# Supplementary material for: Biologically Relevant Micellar Nanocarrier Systems for Drug Encapsulation and Functionalization of Metallic Nanoparticles
Source: Nanomaterials (Basel). 2022 May 20;12(10):1753. doi: 10.3390/nano12101753 (PMC9145561; doi:10.3390/nano12101753)
Supplement: Supplementary file 1 [file nanomaterials-12-01753-s001.zip › nanomaterials-1720112-supplementary.pdf]

nanomaterials

Supplementary Materials

Biologically Relevant Micellar Nanocarrier Systems for Drug Encapsulation and Functionalization of Metallic Nanoparticles

Victoria Valdivia [1,*], Raúl Gimeno-Ferrero [1], Manuel Pernia Leal [1,*], Chiara Paggiaro [1], Ana María Fernández-Romero [2], María Luisa González-Rodríguez [2] and Inmaculada Fernández [1,*]

[1] Departamento de Química Orgánica y Farmacéutica, Facultad de Farmacia, Universidad de Sevilla, 41012 Seville, Spain; rgimeno@us.es (R.G.-F.); chiara.paggiaro@studenti.units.it (C.P.)
[2] Departamento de Farmacia y Tecnología Farmacéutica, Facultad de Farmacia, Universidad de Sevilla, 41012 Seville, Spain; anaferrom2@alum.us.es (A.M.F.-R.); malugoro@us.es (M.L.G.-R.)
* Correspondence: vvaldivia@us.es (V.V.); mpernia@us.es (M.P.L.); inmaff@us.es (I.F.)

Citation: Valdivia, V.; Gimeno-Ferrero, R.; Pernia Leal, M.; Paggiaro, C.; Fernández-Romero, A.M.; González-Rodríguez, M.L.; Fernández, I. Biologically Relevant Micellar Nanocarrier Systems for Drug Encapsulation and Functionalization of Metallic Nanoparticles. *Nanomaterials* **2022**, *12*, 1753. https://doi.org/10.3390/nano12101753

Academic Editor: Stefano Leporatti

Received: 25 April 2022
Accepted: 17 May 2022
Published: 20 May 2022

Publisher's Note: MDPI stays neutral with regard to jurisdictional claims in published maps and institutional affiliations.

Copyright: © 2022 by the authors. Licensee MDPI, Basel, Switzerland. This article is an open access article distributed under the terms and conditions of the Creative Commons Attribution (CC BY) license (https://creativecommons.org/licenses/by/4.0/).

Synthesis of Surfactants

*Synthesis of Compound **3***

Compound **2**

A solution 0.5 M of propargylamine (1 equivalent) and dimethylaminopyridine (0.2 equivalent) in dry CH_2Cl_2 was added to a solution of 0.5 M of oleic acid (0.8 equivalent) and diisopropylcarbodiimide (1.1 equivalent) in dry CH_2Cl_2. The reaction mixture was stirred under argon atmosphere overnight. The reaction was diluted with CH_2Cl_2, and the organic layer was washed with HCl 1M (2 × 10 mL), saturated $NaHCO_3$ aqueous solution (10 mL), and brine (10 mL). The organic layer was dried over anhydrous Na_2SO_4. The solvent was removed, and the crude product was chromatographed (EtOAc: Hexane, 1:5) to yield a white solid **2** (57% purity).

***Rf* (Hexane/EtOAc 3:1)**: 0.55

^{1}H NMR (500 MHz, $CDCl_3$): δ 5.6 (bs, 1H), 5.39–5.35 (m, 2H), 4.1 (m, 2H), 2.25–2.235 (m, 1H), 2.22–2.19 (m, 2H), 2.05–2.01 (m, 4H), 1.67–1.64 (m, 2H), 1.33–1.29 (m, 20H), 0.9 (t, 3H, *J* = 7 Hz).

^{13}C NMR (125.7 MHz, $CDCl_3$): δ 170.4, 155.7, 80.0, 70.7, 70.7, 70.6, 70.0, 68.9, 64.4, 60.3, 53.4, 50.7, 42.4, 28.3.

HRMS calc. for $C_{21}H_{37}NONa$ [M+Na]$^+$: 342.2753, found 342.2767.

Compound **3**

A solution of 1 M of N_3-TEG-NH_2 (1 equivalent) and **2** (1 equivalent) in CH_2Cl_2 was added to a solution of $CuSO_4$ (0.15 equivalent) and sodium ascorbate (0.45 equivalent) in water. The mixture was stirred at room temperature for 3 days. Next, the organic layer was separated and dried over anhydrous Na_2SO_4, and the CH_2Cl_2 was evaporated. The crude product was chromatographed (CH_2Cl_2: MeOH, 9:1) to yield a white solid **3** (30% purity).

***Rf* (CH_2Cl_2/MeOH 9:1)**: 0.08

^{1}H NMR (500 MHz, MeOD): δ 7.8 (s, 1H), 5.39–5.23 (m, 2H), 4.47 (t, *J* = 6, 2H), 4.2 (s, 2H), 3.8 (t, *J* = 5 Hz, 2H), 3.61–3.60 (m, 2H), 3.55–3.50 (m, 8H), 3.0 (s, 2H), 2.12 (t, *J* = 7.5 Hz, 2H), 1.93–1.92 (m, 4H), 1.79–1.73 (m ,2H), 1.51 (m, 2H), 1.22–1.19 (m, 20H), 0.80 (t, *J* = 7 Hz, 3H).
^{13}C NMR (125.7 MHz, MeOD): δ 176.2, 130.9, 130.8, 124.9, 71.5, 71. 4, 71.3, 71.2, 70.4, 68. 2, 51.4, 37.0, 35.5, 33.0, 30.8, 30.5, 30.4, 30.3, 30.3, 30.3, 30.2, 28.1, 28.1, 26.9, 23.7, 14.4.
HRMS calc. for $C_{29}H_{55}N_5O_4$ [M+H]$^+$: 538.43; found 538.43.

*Synthesis of Compounds **4** and **5***

Compound **4**

A solution of 1 M of N_3-TEG-NH_2 (1 equivalent) in dry CH_2Cl_2, under argon atmosphere was added to a solution of Et_3N (1.1 equivalent) and oleoyl chloride (1 equivalent) at room temperature for 24 h. The reaction was diluted with CH_2Cl_2, and the organic layer was washed with HCl 1M (3 × 5 mL), saturated $NaHCO_3$ aqueous solution (5 mL), and brine (10 mL). The organic layer was dried over anhydrous Na_2SO_4. The solvent was removed, and the crude product was chromatographed (CH_2Cl_2: MeOH, 15:1) to yield a white solid **4** (57% purity).

***Rf* (CH_2Cl_2/MeOH 9:1)**: 0.60.
^{1}H NMR (500 MHz, $CDCl_3$): δ 6.0 (s, 1H), 5.35–5.33 (m, 2H), 3.69–3.60 (m, 10H), 3.5 (t, *J* = 5 Hz, 2H), 3.47–3.45 (m, 2H), 3.39 (t, *J* = 5 Hz, 2H), 2.17 (t, *J* = 7.5 Hz, 2H), 2.02–1.98 (m, 4H), 1.64–1.61 (m, 2H), 1.30–1.25 (m, 20H), 0.89 (t, *J* = 6.5 Hz, 3H).
^{13}C NMR (125.7 MHz, $CDCl_3$): δ 173.4, 130.1, 129.9, 70.9, 70. 8, 70.7, 70.4, 70.2, 70.1, 50.8, 39.3, 36. 9, 32.0, 29.9, 29.9, 29.8, 29.7, 29.5, 29.3, 27.4, 27.3, 25.9, 22.8, 14.2.
HRMS calc. for $C_{26}H_{50}N_4O_4Na$ $[M+Na]^+$: 505.3724; found 505.3718

Compound **5**

To a solution 0.2 M of the azide **4** (1 equivalent) in dry THF was added at 0°C a 1 M LAH solution in THF under argon atmosphere. After 1 h, the reaction mixture was quenched with saturated Na_2SO_4 aqueous solution, and it was stirred for 30 min at room temperature. The mixture was filtered over celite and washed with ether (5 × 10 mL) and then with CH_2Cl_2 (10 mL). The combined organic phases were dried over anhydrous Na_2SO_4, filtered and concentrated. The crude product was chromatographed (CH_2Cl_2: MeOH, 15:1) to yield a colorless oil **5** (65% purity)

***Rf* (CH_2Cl_2/MeOH 15:1) =** 0.1
^{1}H NMR (500 MHz, $CDCl_3$): δ 6.6 (s, 1H), 5.35–5.32 (m, 2H), 3.65–3.56 (m, 10H), 3.48–3.44 (m, 2H), 3.04–3.03 (m, 2H), 2.98–2.96 (m, 2H), 2.94–2.92 (m, 2H), 2.20–2.17 (m, 2H), 2.02–1.98 (m, 4H), 1.63–1.60 (m, 2H), 1.30–1.27 (m, 20H), 0.88 (t, *J* = 7 Hz, 3H).
^{13}C NMR (125.7 MHz, $CDCl_3$): δ 173.7, 130.1, 129.9, 70.6, 70.5, 70.3, 70.2, 39.3, 36.8, 32.0, 29.9, 29.9, 29.8, 29.81, 29. 7, 29.5, 29.4, 29.3, 27.4, 27.3, 25.9, 22.8, 14.2.
HRMS calc. for $C_{26}H_{52}N_2O_4$ $[M+H]^+$: 457.71; found 457.40

NMR Spectra

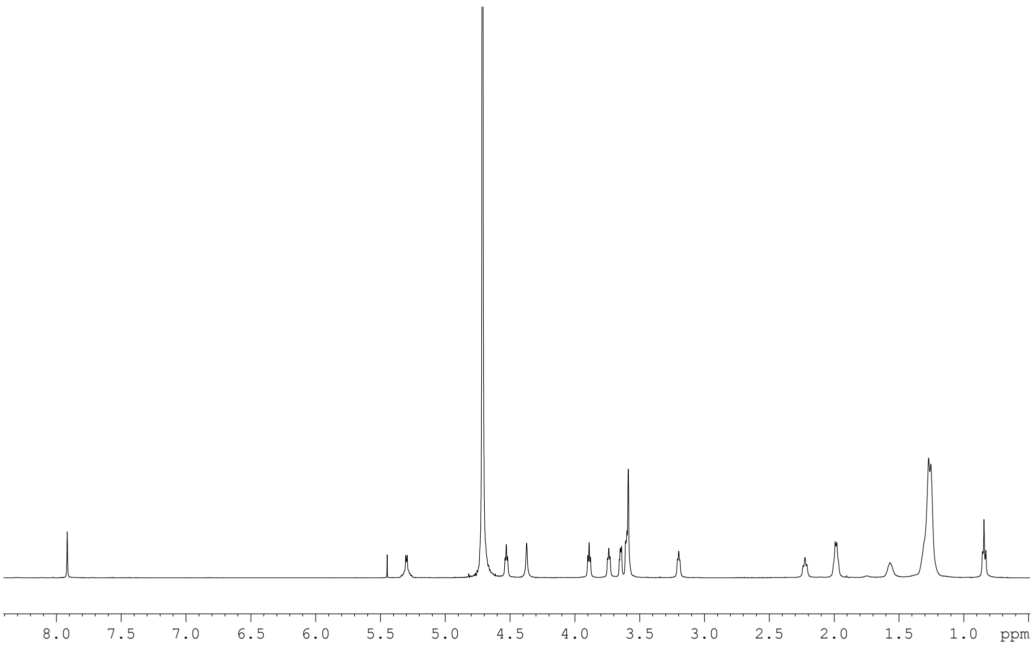

Figure S1. ^{1}H-NMR of 3 in D_2O.

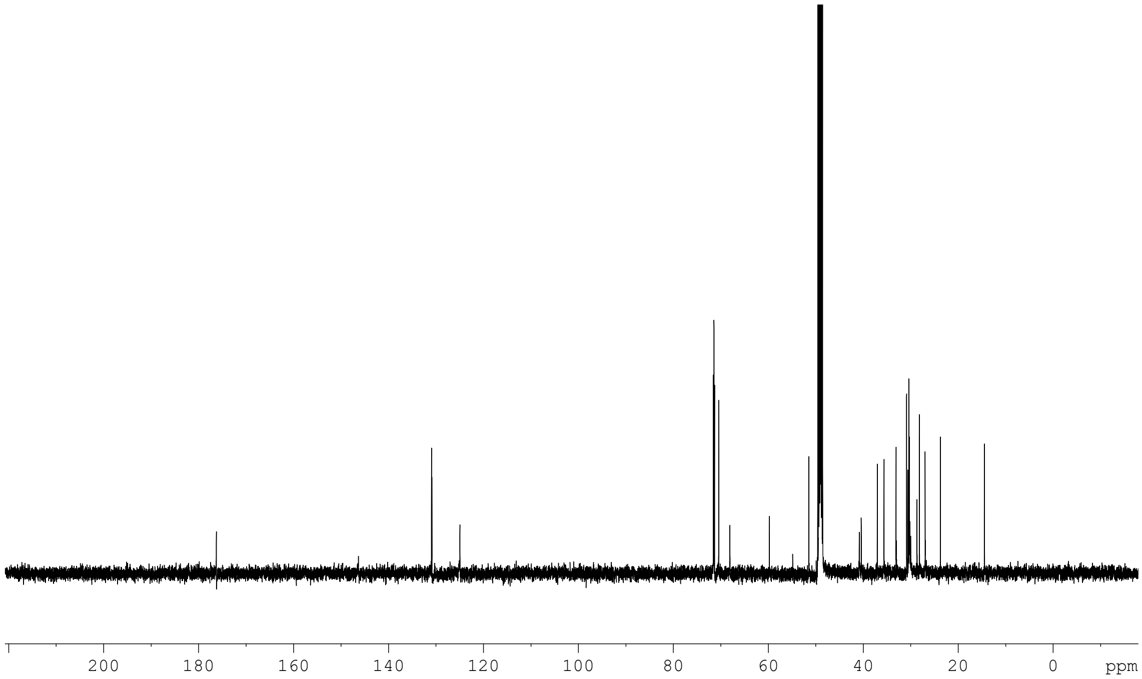

Figure S2. ^{13}C-NMR of 3 in MeOD.

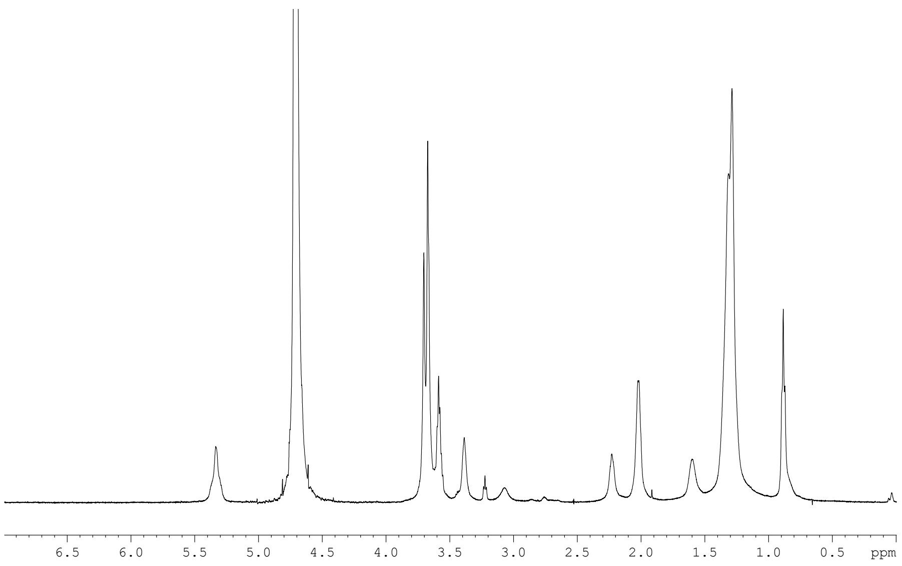

Figure S3. ^{1}H-NMR of 5 in D_2O.

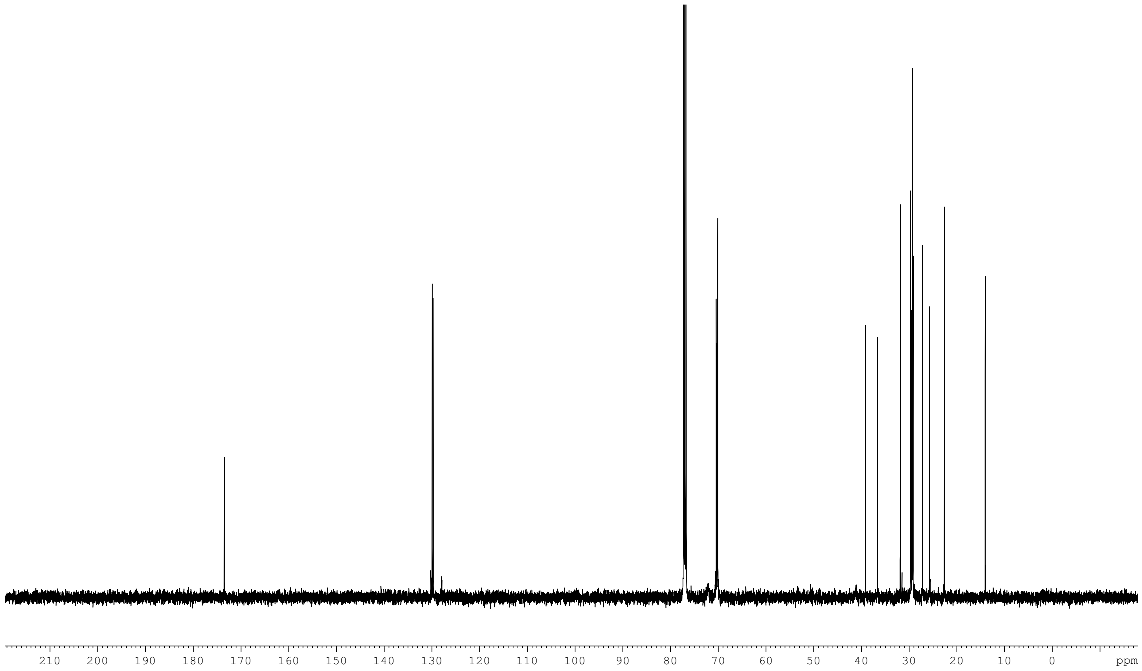

Figure S4. ^{13}C-NMR of 5 in MeOD.

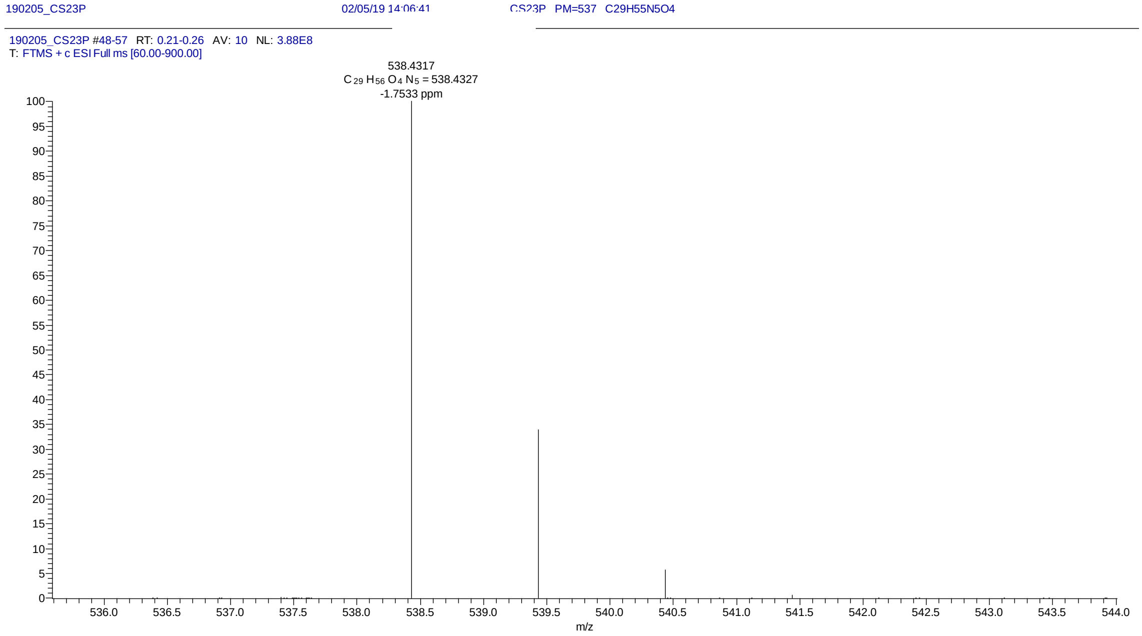

Figure S5. HRMS Spectrum of 3.

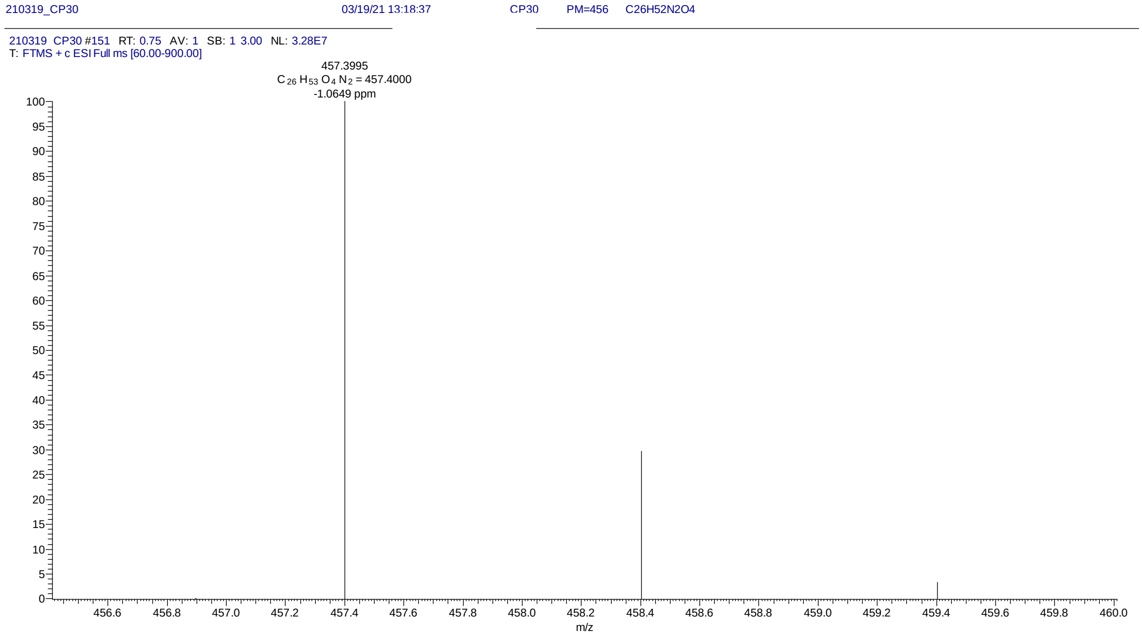

Figure S6. HRMS Spectrum of 5.

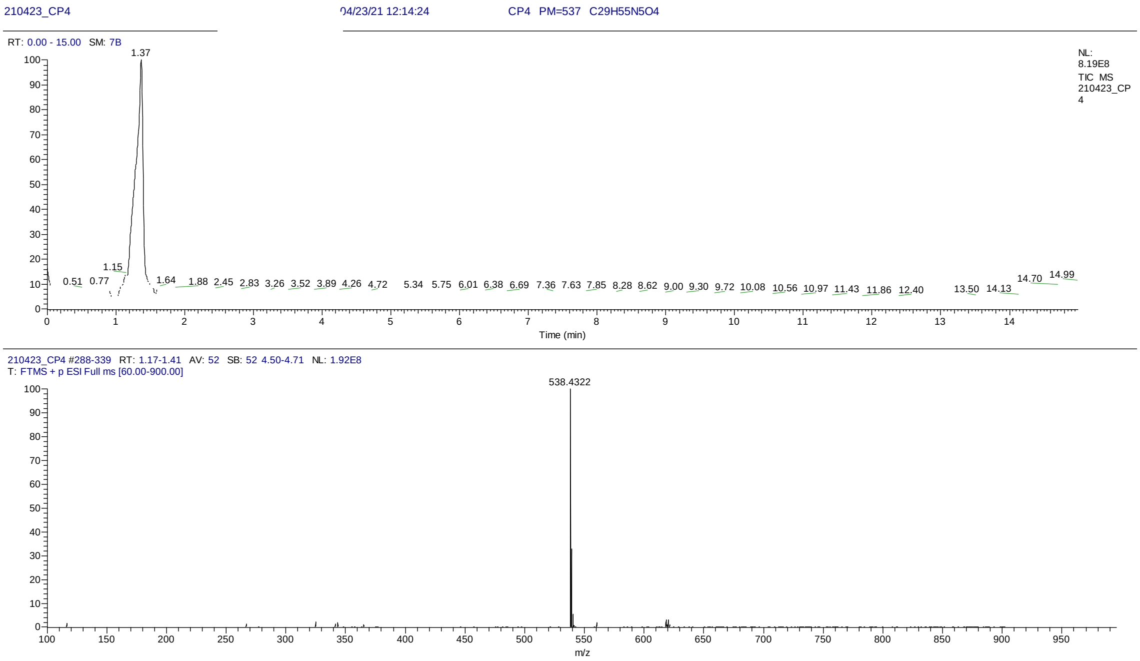

Figure S7. Top graph corresponds to HPLC chromatogram of 3. Bottom graph corresponds to MS Spectrum of 3.

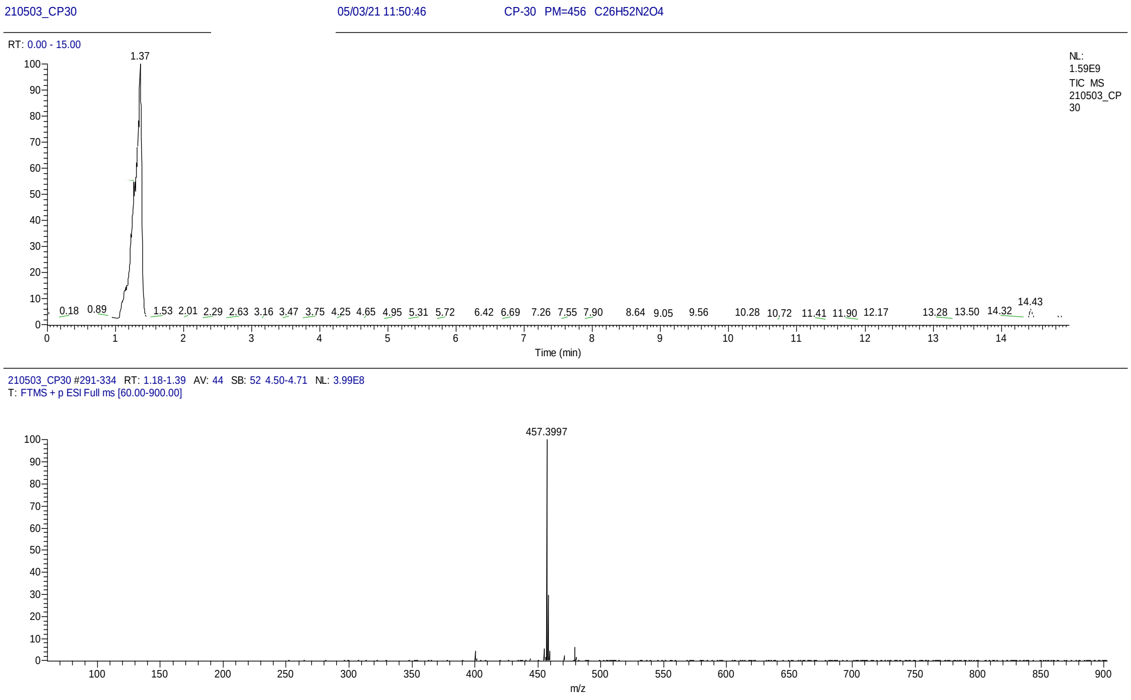

Figure S8. Top graph corresponds to HPLC chromatogram of 5. Bottom graph corresponds to MS Spectrum of 5.

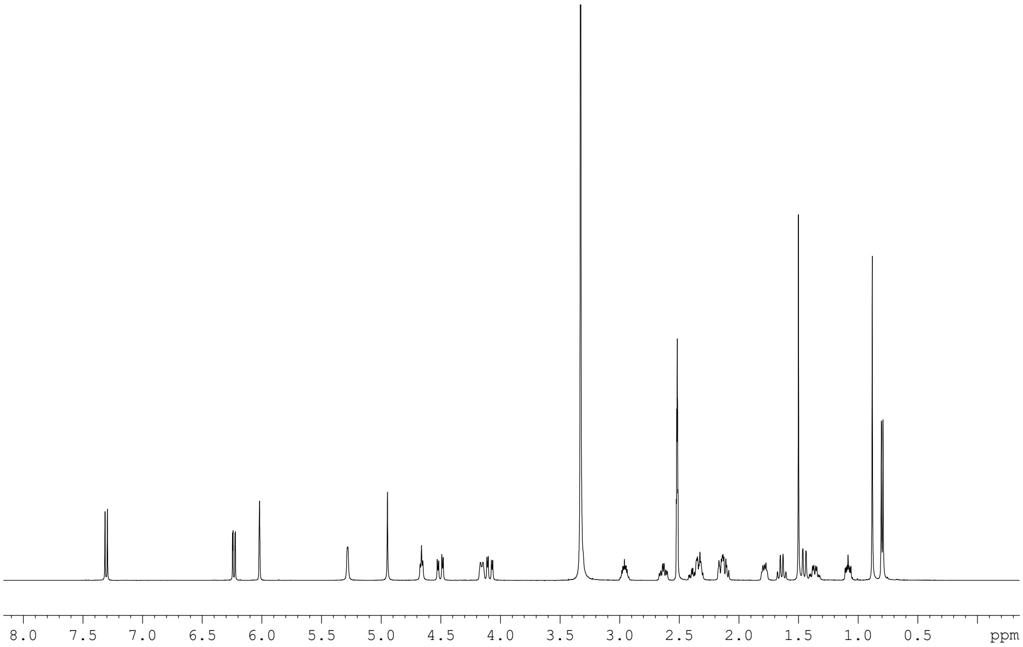

Figure S9. ^{1}H-NMR of dexamethasone in DMSO-d_6.

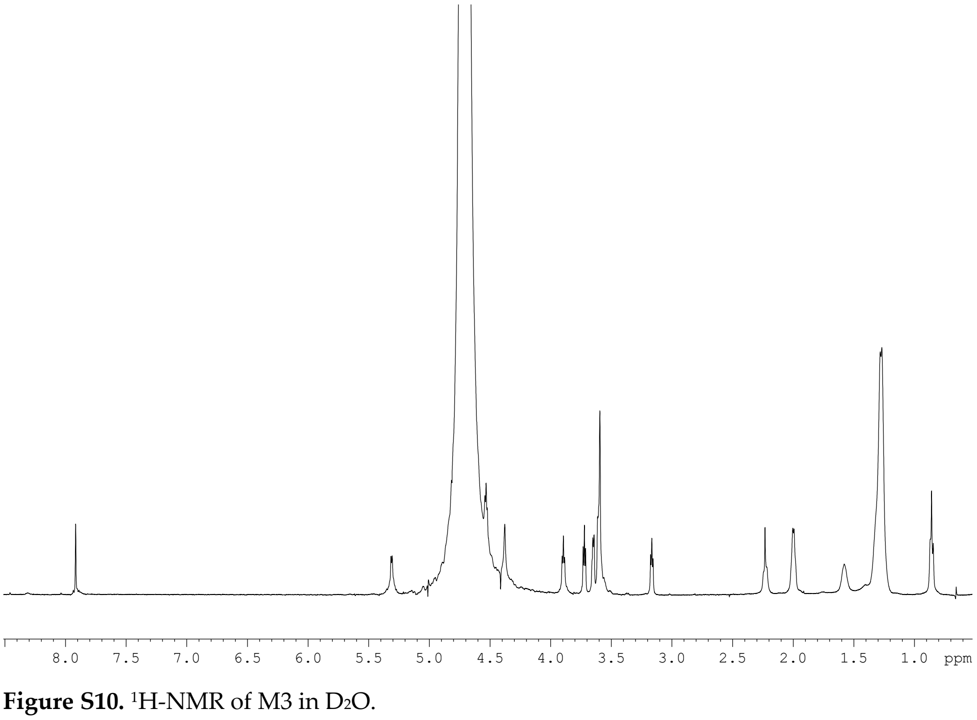

Figure S10. ^{1}H-NMR of M3 in D_2O.

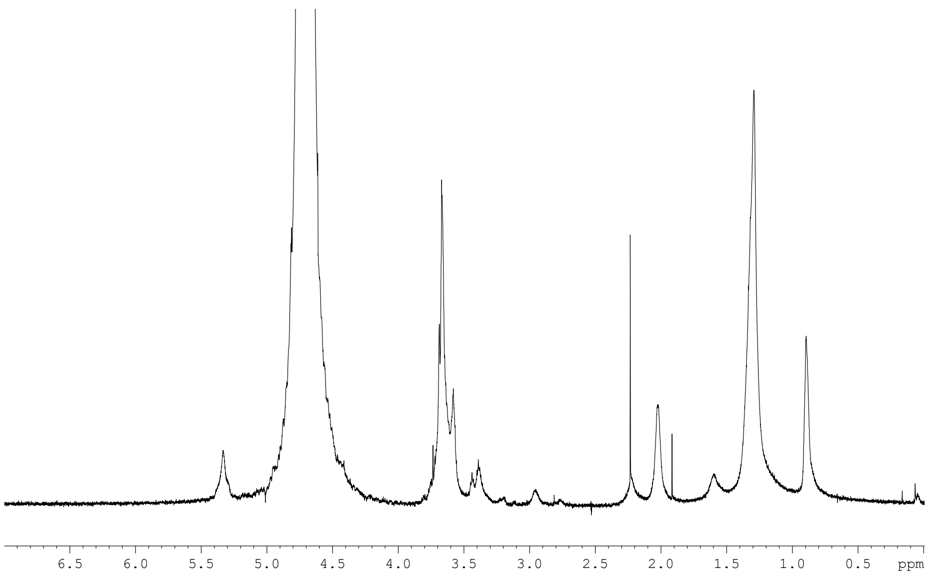

Figure S11. ^{1}H-NMR of M5 in D_2O.

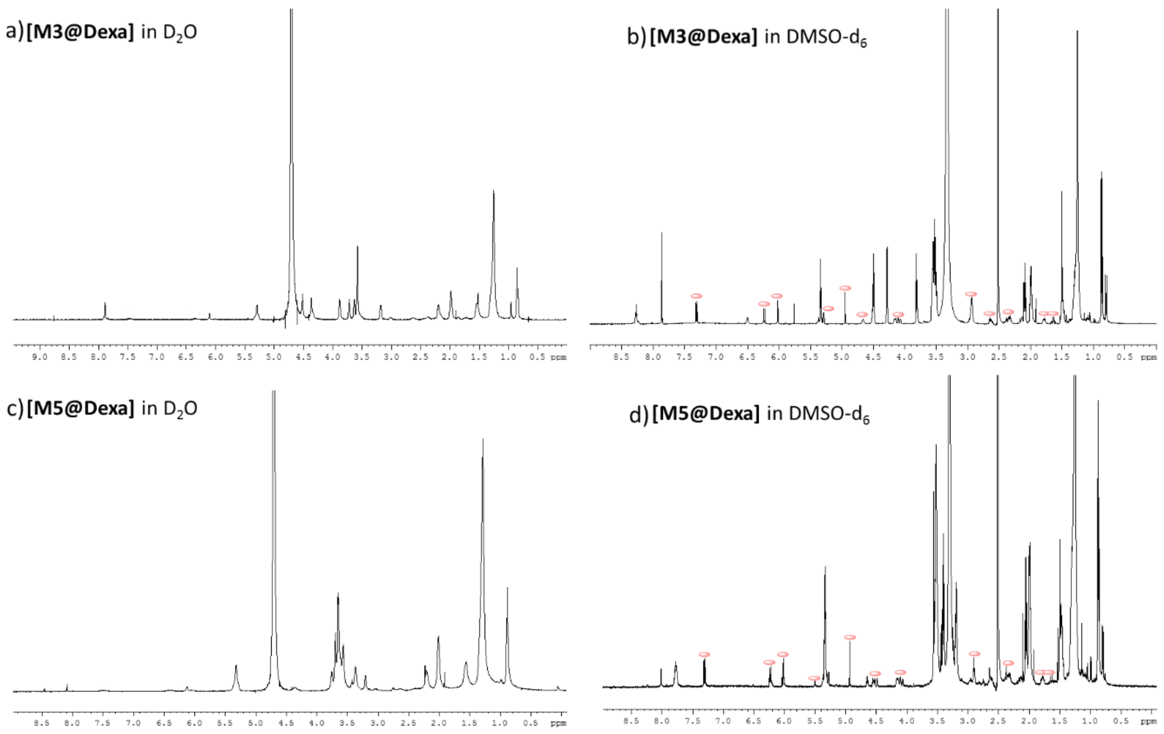

Figure S12. ^{1}H-NMR of **[M3@Dexa]** in D_2O (**a**) and in DMSO-d_6 (**b**). ^{1}H-NMR of **[M5@Dexa]** in D_2O (**c**) and in DMSO-d6 (**d**). In pink are indicated the new signals that appear in DMSO-d6 ((**b**) and (**d**)) corresponding to dexamethasone.

CMC Curve of Compounds 3 and 5

CMC values were determined using pyrene as an extrinsic fluorescence probe. The pyrene concentration was maintained constant (0.6 10^{-6}M in THF), and the concentration of **3** or **5** was varied from 10^{-3} M to 10^{-6}. Fluorescence measurements were carried out at 25 °C using a Varian Cary Eclipse spectrofluorometer. At the fixed excitation wavelength of 334 nm, the emission spectra were scanned from 300 to 350 nm. The fluorescence intensity ratios of pyrene at 339 and 335 nm (I339/I335, I1/I3) were calculated and plotted against the concentration logarithm of the amphiphilic compound. The CMC value was determined from the intersection of the two tangent lines.

Sample	Concentration C	Log C	I1/I3
1	0.000236997	−3.62525756	0.4349
2	0.000157998	−3.801348819	0.4348
3	7.89989 x 10^{-5}	−4.102378815	0.4337
4	3.94995 x 10^{-5}	−4.403408811	0.4642
5	1.97497 x 10^{-5}	−4.704438806	0.4813
6	9.87487 x 10^{-6}	−5.005468802	0.4886
7	4.93743 x 10^{-6}	−5.306498798	0.5402

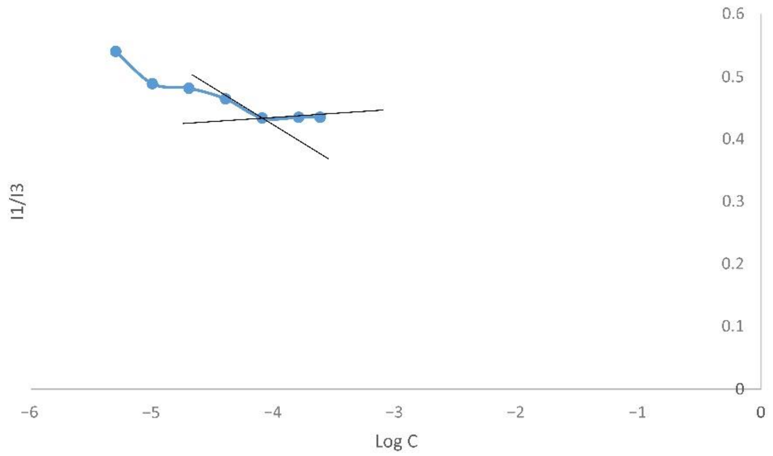

Figure S13. CMC curve of Compound **3.**

Sample	Concentration C	Log C	I1/I3
1	0.00034528	−3.461828577	1.1371
2	0.00020717	−3.683673134	1.2745
3	0.00017264	−3.762858573	1.2734
4	6.90551 x 10^{-5}	−4.160804552	1.3055
5	3.45275 x 10^{-5}	−4.461834547	1.3714
6	1.72638 x 10^{-5}	−4.762864543	1.3883
7	8.63188 x 10^{-6}	−5.063894539	1.395
8	4.31594 x 10^{-6}	−5.364924534	1.4229
9	2.15797 x 10^{-6}	−5.66595453	1.4939

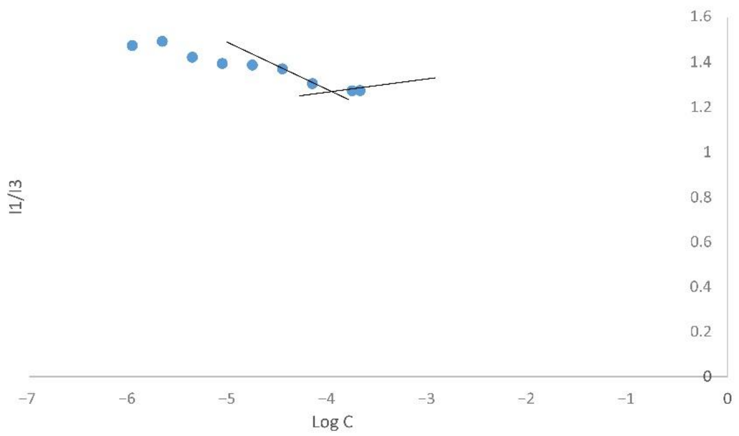

Figure S14. CMC curve of Compound **5.**

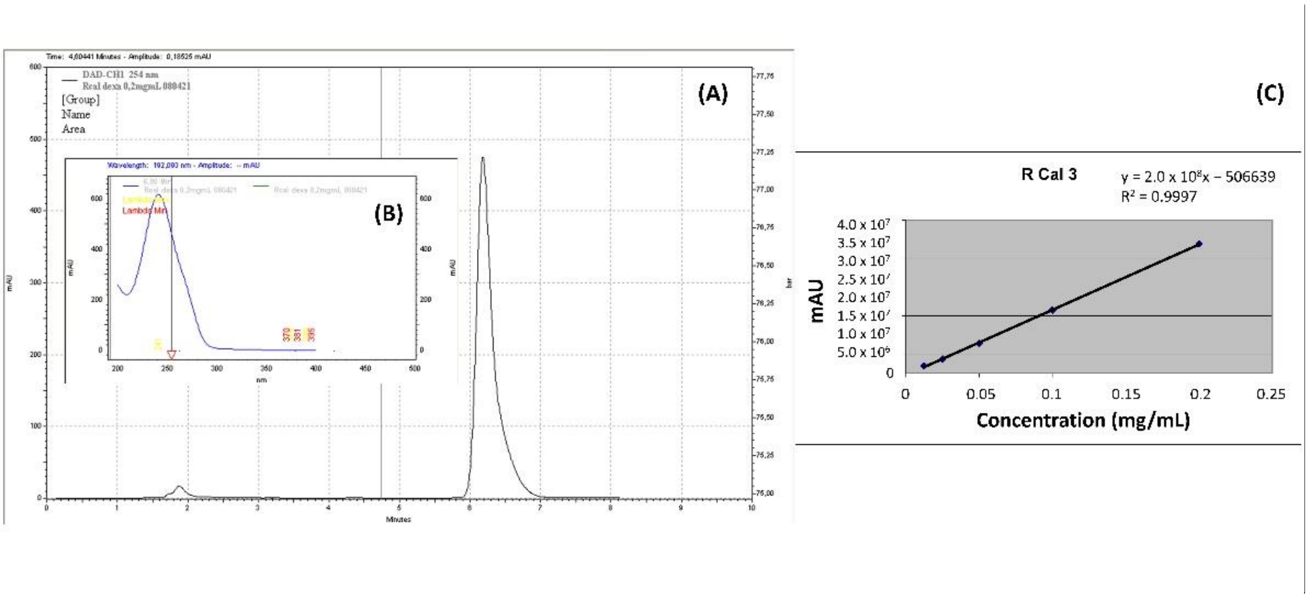

Figure S15. (**A**) HPLC chromatogram of Dexa, (**B**) UV absorption spectrum of Dexa, and (**C**) Calibration curve of Dexa.

Release Procedure of Dexa from M3@Dexa and M5@Dexa

M3@Dexa or **M5@Dexa** (2 mg) were transferred into a dialysis bag containing 4 ml of milliQ water at pH 7.4. The bag was closed at both ends with plastic clamps and tested for leakage. This bag was then placed into 300 mL of milliQ water at pH 7.4 and gently stirred for 52 h at room temperature. The bag was fully immersed under the surface of the liquid. Aliquots of about 2 ml were withdrawn from the 300 mL recipient at specified time periods. The withdrawn aliquots were replaced with equal volumes of milliQ water. Aliquots were then filtered using a 45 μm syringe filter. Aliquots were analyzed using HPLC.

Stability Study of Micelles in Water vs. Time

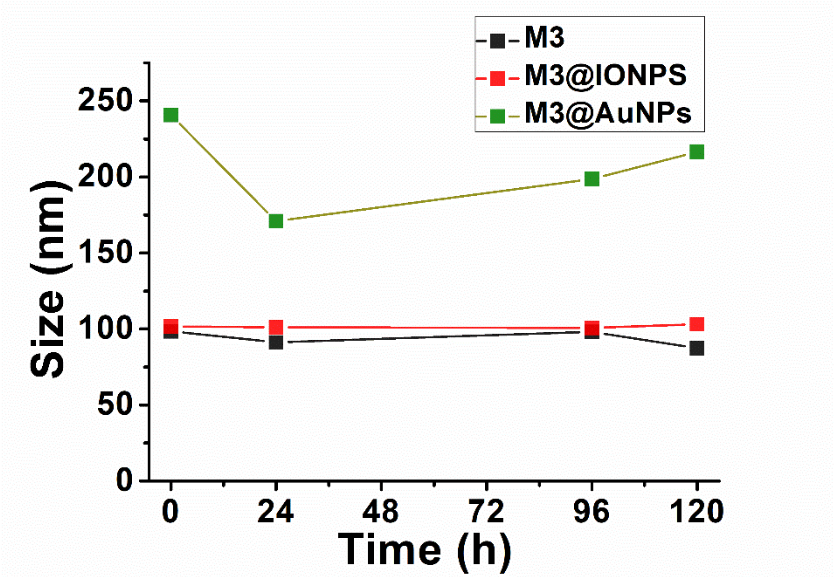

Figure S16. Hydrodynamic sizes of micelles **M3**, **M3@IONPs** and **M3@AuNPs** in water vs. time.
